# A qualitative exploration of Malaysian cancer patients’ perceptions of cancer screening

**DOI:** 10.1186/1471-2458-13-48

**Published:** 2013-01-18

**Authors:** Maryam Farooqui, Mohamed Azmi Hassali, Aishah Knight, Asrul Akmal Shafie, Muhammad Aslam Farooqui, Fahad Saleem, Noman-ul Haq, Hisham Aljadhey

**Affiliations:** 1 Faculty of Pharmacy, Universiti Teknologi MARA (UiTM), Penang, Malaysia; 2 Discipline of Social and Administrative Pharmacy, School of Pharmaceutical Sciences, Universiti Sains Malaysia, Penang, Malaysia; 3 Advanced Medical and Dental Institute, Universiti Sains Malaysia, Penang, Malaysia; 4 Department of Medicine, Allianze University College of Medical Sciences (AUCMS), Penang, Malaysia; 5 Department of Pharmacy, University of Baluchistan, Quetta, Pakistan; 6 College of Pharmacy, King Saud University, Riyadh, 11451, Saudi Arabia

**Keywords:** Cancer, Screening, Perceptions, Malaysia, Qualitative method

## Abstract

**Background:**

Despite the existence of different screening methods, the response to cancer screening is poor among Malaysians. The current study aims to examine cancer patients’ perceptions of cancer screening and early diagnosis.

**Methods:**

A qualitative methodology was used to collect in-depth information from cancer patients. After obtaining institutional ethical approval, patients with different types and stages of cancer from the three major ethnic groups (Malay, Chinese and Indian) were approached. Twenty semi-structured interviews were conducted. All interviews were audiotaped, transcribed verbatim, and translated into English for thematic content analysis.

**Results:**

Thematic content analysis yielded four major themes: awareness of cancer screening, perceived benefits of cancer screening, perceived barriers to cancer screening, and cues to action. The majority of respondents had never heard of cancer screening before their diagnosis. Some participants reported hearing about mammogram and Pap smear tests but did not undergo screening due to a lack of belief in personal susceptibility. Those who had negative results from screening prior to diagnosis perceived such tests as untrustworthy. Lack of knowledge and financial constraints were reported as barriers to cancer screening. Finally, numerous suggestions were given to improve screening behaviour among healthy individuals, including the role of mass media in disseminating the message ‘*prevention is better than cure*’.

**Conclusions:**

Patients’ narratives revealed some significant issues that were in line with the Health Belief Model which could explain negative health behaviour. The description of the personal experiences of people with cancer could provide many cues to action for those who have never encountered this potentially deadly disease, if incorporated into health promotion activities.

## Background

Cancer is a leading cause of death worldwide. The World Health Organization (WHO) estimated that there were 7.6 million deaths due to cancer in 2008 and this number is likely to rise to 13.1 million deaths by the year 2030 [1,2]. Until 2001, Malaysia lacked a National Cancer Registry (NCR) to estimate the incidence of cancer among Malaysians [3]. Data published in the first national report on cancer incidence among Malaysians in 2002 and in the most recent in 2006 revealed that the number of new cancer cases among residents in peninsular Malaysia has decreased from 26,089 in 2002 to 21,773 in 2006 [4]. However, when unregistered cases are taken into account the risk that a Malaysian may be expected to get cancer in his/her lifetime can be estimated as 1 in 4 [5,6]. Although there is a decline in numbers of new cases, presentations at the late stages are still on the rise [7,8]. Lifestyle factors such as lack of regular exercise, the consumption of a high fat diet, and breastfeeding habits have been reported as some of the risk factors among Malaysian breast cancer patients [9]. The incidence of cervical cancer is increasing gradually [10] and both breast and cervical cancer are among the leading causes of death for Malaysian female cancer patients. The incidence of colon cancer is reported to be rising worldwide; this may be due in part to lifestyle factors such as dietary habits and smoking, which doubles the risks of colon cancer [11,12]. Among Malaysians, the highest incidence of colon cancer is found in the Chinese population, followed by the Malay and Indian populations [13].

Within this context, the early detection of cancer can greatly increase the chances of survival. Cancers of the breast, cervix, colorectum, and prostate are among the few cancers which can be detected via screening techniques. Mammography, Breast Self Examination (BSE), and Clinical Breast Examination (CBE) are some of the effective methods for early detection of breast cancer. Socio-demographic factors such as age and educational status are identified as barriers to BSE among Malaysian women workers [14]. Kanaga et al. (2011) reported that 92.8%, 50.4% and 47.2% of Malaysian women in their study correctly answered questions on capability of BSE, CBE and mammography, respectively [15]. The same study concluded that awareness of breast cancer and practice of screening procedures increases with higher education and urban living.

Pap smear screening was first introduced in Malaysia in 1960; which mainly involved women who attended the medical facilities during the antenatal and postnatal check-ups [16]. Though organized screening programmes may be more effective than opportunistic screening, Malaysia is highly reliant on opportunistic screening [17]. However, the results of such programmes have not been satisfactory for reducing the incidence of cervical cancer among Malaysia women due to individual factors and the weakness in the health screening system [18]. Parsa et al. (2008) in a review of literature concluded that lack of knowledge of breast cancer and cancer screening methods, cultural attitudes, modesty, lack of encouragement by family members and physicians as major reasons for the poor response to cancer screening [19]. Wong et al. (2009) reported that many of the women in their study believed that the purpose of the Pap smear test is to detect existing cervical cancer, and thus Pap smear screening is not required until a woman appears with clear symptoms of cervical cancer [20]. In these studies it is evident that lack of knowledge and differences in health beliefs may affect an individual’s health seeking behaviour.

Several health behaviour models have been proposed to understand an individual’s health-related behaviour. The best known model in public health is the Health Belief Model (HBM) which was first introduced in early 1950s to help understand human behaviour towards seeking health services such as immunization and screening [21]. The model’s four key components are conceptualized as *perceived susceptibility, perceived severity, perceived benefits*, and *perceived barriers*. *Cues to action* is another construct of HBM which helps in the understanding of actions that trigger human behaviour [22]. The model assumes that an individuals’ health behaviour depends upon the belief about the impact of the illness and its consequences, provided that the individual has a distinct course of action by which to proceed. It is important to understand how vulnerable a person considers him- or herself to be to getting a disease, how serious the disease symptoms are for an individual, and how beneficial the suggested course of action is considered to be. A lack of emphasis on these factors may contribute to a poor response to the screening programmes. Though different studies have evaluated the perceptions of healthy Malaysians regarding cancer screening, the current study aimed to evaluate Malaysian cancer patients’ perceptions of cancer screening and their knowledge and experiences of the cancer screening tests.

## Methods

### Research ethics

Ethical approval was obtained from the Medical Research and Ethical Committee of the Malaysian Ministry of Health prior to the commencement of the study. Informed consent was also obtained in either the English or Malay languages from each respondent. Verbal consent was accepted from patients unable to read or write.

### Design and settings

The study was conducted at Penang General Hospital, which provides oncology services for the State of Penang and for the neighbouring states as well. As discussed earlier, many studies have considered healthy Malaysians’ perceptions of cancer screening, the experiences and perceived effectiveness of screening tests among patients with cancer were evaluated in this study through a qualitative approach [23]. Qualitative methods elaborate the understanding of how and why people behave as they do. In addition, these methods provide comprehensive answers to questions. The flexible nature of the exploration is advantageous to the researcher investigating barriers and to the facilitators inviting a particular response [24].

### Participants

The participants were invited from the three major ethnic groups in Malaysia (Malay, Chinese and Indians). Patients aged 18 years and above with a confirmed diagnosis of any type and stage of cancer were included. Recruitment was undertaken by approaching patients in the oncology ward directly after getting permission from the hospital authorities. The only exclusion criterion was cognitive impairment either as a result of cancer or another disease process such as dementia.

### Study tool

A semi-structured interview guide (Table 1) was used as the study tool. The interview guide was developed after an extensive literature search [25,26]. The first draft was discussed among the experts from the School of Pharmaceutical Sciences, Universiti Sains Malaysia. At the same time discussions with public health experts and oncologists were carried out to identify the related issues from the health-care providers’ perspective. Pre-testing of the interview guide was performed but data from the pilot study was not added to the final analysis.

**Table 1:** Interview guide

**Discussion topics**	**Examples of specific probes**
***Awareness towards cancer screening***	Before your diagnosis (cancer), have you heard of cancer screening?
***Perceived benefits of cancer screening***	Do you think cancer screening is useful for early detection?
***Barriers to cancer screening***	What stops people in seeking cancer screening?
***Cues to action***	What would you like to suggest improving cancer screening behaviour among healthy individuals?

### Interview process and data evaluation

Interviews were conducted in the Malay language; however, the Tamil and English languages were used for patients who preferred to communicate in these languages. Two research assistants of Indian and Malay ethnic backgrounds were trained to conduct the interviews. Chinese patients were interviewed either in the Malay or English languages by the same interviewers. Each interview lasted for about 30–60 min. The principal investigator attended all the interviews with the research assistants to take field notes and to facilitate the interview process. Patients’ demographic and disease-related data was obtained by a questionnaire attached to the patient information sheet. All interviews were audiotaped so that verbatim transcriptions could be created. Each interview was transcribed verbatim by the research assistants trained for this purpose. The transcripts were than verified for their accuracy by the principal investigator who listened to the tapes, and they were sent to the participants for approval. Each transcript was read by the principal investigator who recorded the raw data thematically. The themes were then discussed with other independent researchers to ensure their reliability and trustworthiness [27]. Each transcript was repeatedly read to identify the common themes. All authors discussed the emergent themes to refine the analysis [27]. The interviews were continued and not concluded until theoretical saturation was reached when no new information was being produced by subsequent interviews [28].

## Results

Twenty cancer patients (P1–P20) between the ages of 18–70 years (mean = 53 years) were interviewed. The cohort of participants was dominated by Malays (n = 10), followed by Indians (n = 6) and Chinese (n = 4). The majority of the participants were from the low income group seeking treatment in the government hospitals. With one exception, none of the patients had medical insurance. More than half of the participants (n = 16) reported their cancer stage as being from stage II/III (*locally advanced)* to stage IV (*metastasized to other organs*). The demographic and disease-related data are summarized in Table 2. During the analysis, four themes were identified: awareness of cancer screening, perceived benefits of cancer screening, perceived barriers to cancer screening, and cues to action. All the patients were asked about their cancer screening practices before the cancer diagnosis. A majority of the patients had undergone no cancer screening tests before their cancer diagnosis.

**Table 2:** Demographic and clinical characteristics of participants

**Characteristics**	**n**	**%**
***Age range***		
18-30	1	5
31-40	5	25
41-50	5	25
51-60	6	30
61-70	3	15
***Gender***		
Male	7	35
Female	13	65
***Race or Ethnicity***		
Malay	10	50
Indians	6	30
Chinese	4	20
***Education***		
Primary	8	40
Secondary	8	40
Matriculation/Diploma	4	20
***Socioeconomic status***		
Low (Less than RM*1000/month)	10	50
Middle (RM 1000-RM 3000/month)	4	20
High (RM 3100 and above/month)	6	30
***Tumor Site***		
Naso-pharynx	3	15
Colorectal	5	25
Breast	5	25
Cervix	5	25
Liver	1	5
Non-Hodgkin’s Lymphoma	1	5
***Tumor Stage***		
Stage 0 (In situ)	1	5
Stage I (Localized to one part)	3	15
Stage II & III (Locally advanced)	8	40
Stage IV (Metastasized to other organs)	8	40

*RM* Ringgit Malaysia.

### Awareness of cancer screening

When the participants were asked if they have heard of cancer screening before the cancer diagnosis the majority denied hearing about or seeking cancer screening tests. Male cancer patients, who were aware of breast cancer screening, never thought of encouraging their female family members to go for screening:

*I don’t know about cancer screening. I heard about breast cancer but since it wasn’t of interest to me, I ignored it….* (P11)

However, female participants reported hearing about mammograms and Pap smear tests:

*I read about the mammogram and Pap smear.* (P2)

At the same time, the belief that a healthy person does not have to see the doctor constrained some of the patients from seeking screening tests, which indicates a poor level of knowledge regarding cancer screening:

*When we are healthy and go to see the doctor just for fun, it will be embarrassing, right?* (P17)

### Perceived benefits of cancer screening

There were mixed beliefs about the effectiveness of screening for early detection:

*I think screening would be better in order to know about it* [cancer] *but if it is late.. no point.* (P18)

*Yeah … Screening can be useful, but six months before the diagnosis of cancer, I went for ultrasound and an endoscopy … still they couldn’t detect cancer, even though I was down with anaemia, but the ultrasound couldn’t detect it. Only at a later stage with the CT scan did they find out I had got cancer, so screening sometimes also doesn’t show it,*[there is no point in screening] *except if there is a very effective way to detect cancer earlier.* (P6)

### Perceived barriers to cancer screening

Lack of information about cancer screening was recognised as a major barrier for the cohort in the current study:

*I don’t think they* [doctors] *are giving sufficient information to the public about cancers, now also they are only focusing on breast cancer.* (P2)

The language in which most of the cancer screening awareness material is disseminated to the public was identified as a barrier to cancer screening:

*I never read about cancer screening before the diagnosis. I just read posters at hospitals which are usually in the Malay language. It should be written in other languages too, like Tamil or Mandarin. Sometimes it’s in English which I don’t understand either*. (P4)

Financial constraint was another important obstacle for patients:

*The doctor suggested a pap smear to confirm the problem. I refused to do so because I need to pay on my own.* (P17)

The patients’ narratives revealed that a poor level of perceived susceptibility to cancer was another major barrier to patients seeking cancer screening tests. Patients with diabetes or hypertension reported going for regular health check-ups but they never perceived that they might have cancer and thus they never thought of asking for screening tests:

*Actually I am a medical lab technologist so I always hear about screening and tests, stuff like this, but I never thought of having one* [screening] *for me as I was always healthy.* (P12)

Even patients with a strong family history of cancer never perceived themselves as being at risk of cancer:

*Yes, my father at the age of 63, and my younger sister at the age of 20 years died of stomach cancer, but I never thought of getting cancer, because I was healthy all the time.* (P6)

At the same time, fatalistic beliefs about cancer and its diagnosis stopped patients going for screening:

*Yeah. I have heard of it* [Pap smear] *before, but I just ignored it … as everything comes from Allah* [God in Muslim belief]*.* (P18)

### Cues to action

The need for the mass media to play its part in disseminating information about the early detection of cancer and screening programmes was an issue raised by most of the patients. Patients urged the information industry, specifically television and radio to play its part effectively:

*We should have more programmes on radio and TV as well as celebrities talking about early cancer detection. Like one American cookery show, the host always relates the food with cancer, like what to eat and what not to eat to avoid cancer; we should also have such programmes in local languages*. (P2)

*I think we need to provide education, come up with articles in a simple language, and make it available everywhere for them* [people] *to read, not only in the hospitals but in public places too.* (P12)

Introducing screening programmes at work places at a discounted rate and at a feasible time was another useful idea proposed by the patients:

*They* [authorities for cancer screening] *should go into the factory as usually there will be a sick bay inside. They can offer Pap smears for free. They offer it for free in the government clinics, right? That is the only way we can help others* (P17)

## Discussion

The aim of this study was to explore cancer patients’ perspectives of cancer screening. The study population was diverse in terms of its ethnicity and the stage and type of cancers. The results highlight the necessity of disseminating information about cancer screening to healthy individuals through more culturally specific approaches. Awareness of cancer screening and its potential benefits in terms of early detection was insufficient. The majority of the patients reported that they had never heard of cancer screening before their diagnosis. Though subsidized mammogram and Pap smear screening tests are available at most government health clinics, none of the female cancer patients had received those tests prior to the diagnosis. Othman and Rebolj (2009) in a review of literature concluded that despite offering Pap smear tests free of charge since 1995, only 47.5% Malaysian women were screened for cervical cancer [29]. The same study identified awareness of the disease and its prevention as important determinants of reducing cervical cancer burden in Malaysia.

During the analysis several constructs of health belief, such as perceived susceptibility to getting cancer, perceived benefits of cancer screening tests, and perceived barriers to cancer screening were identified to help explain Malaysians’ apparent reluctance to undergo cancer screening. Perceived susceptibility, or a person’s view of how vulnerable he or she is to a disease, can influence that person’s attitude to taking certain actions [21]. The study participants who had heard of cancer screening before the diagnosis never perceived themselves as being at risk of getting cancer. This coincides with the findings of a study which concluded that perceived susceptibility to cervical cancer predicts women’s cervical cancer screening behaviour [20].

The Health Belief Model also describes knowledge of the illness as a modifying factor in the utilization of health care services [30]. Fatalistic beliefs towards cancer prohibited some of the participants in seeking screening tests. As published previously, the same study population reported the causes of cancer as being God’s will, dietary factors, and unhealthy life style [31]. Such understating of cancer clearly indicates a poor level of knowledge, and this can affect the utilization of screening tests available for early detection. Thus, efforts to improve screening behaviour should focus on removing fatalistic beliefs about cancer and providing factual information on the importance of cancer screening and its potential benefits in reducing the incidence of cancer diagnosis at advanced stages.

The perceived benefits of preventive health practices influence a person’s willingness to take preventive measures [21]. Although the participants recognized the importance of screening for early detection, concerns were raised about the effectiveness of these tests for early detection. The Faecal Occult Blood Test (FOBT) and sigmoidoscopy together with colonoscopy are recommended methods for the early detection of colon cancer [32]. When introducing any campaign for cancer screening, emphasis should be placed on proven methods of screening because high numbers of false positives or false negatives leads to a lack of confidence in the screening programme. When opportunistic screening programmes are implemented, it is essential that health-care providers are made aware of the early signs of cancers and can thus ensure patients undergo effective screening methods.

The basic health-seeking behaviour models have identified several physical and psychological barriers, which may affect an individual’s ability to utilize health-care services [21,22,33]. During the discussion, various barriers were identified which could explain the poor response to cancer screening. Lack of awareness about cancer screening tests was identified as a major barrier to screening. Although tremendous efforts have been made by government and non-governmental organizations on early detection, the lack of collaborative care and flow of information affects patients’ knowledge of what is happening and what is available. At the same time, the nature of a disease where an individual remains asymptomatic at the early stages hinders patients from seeking screening tests. In terms of affordability, in some cases screening tests were advised by the doctors but patients refused them due to their inability to pay. However, doctors must be aware of different alternatives such as public hospitals and other public health services where screening tests such as the Pap smear for cervical cancers and the mammogram for breast cancers are available at subsidized rates [34]. Beside these barriers, Abdullah et al. (2011) identified younger age, never been pregnant, lower socioeconomic status and lack of health insurance coverage as factors associated with poor response to Pap smear screening test [35]. Considering socio-economic status as a barrier to cancer screening, it is important to highlight that all of the participants of this study was from low to middle income group which might be a reason to poor response to cancer screening tests.

Cues to action is another component of the Health Belief Model which can provide strategies for promoting awareness by identifying the factors that influence human action. The role of the mass media in disseminating and improving awareness of cancer was highlighted by the patients. Patients wanted local celebrities to encourage people to come forward for screening. Since Malaysia is a multi-ethnic country, emphasis should be placed on the local languages specific to different ethnic groups for disseminating information. During the interviews we also identified that the Malay language was not commonly used by older Indian and Chinese patients who are at higher risk of developing cancer. Keeping this in view, health awareness campaigns should be more culturally specific to different ethnic groups. At present many government hospitals provide information leaflets in languages other than Malay including Tamil and Mandarin. Thus these information leaflets can be made available to other public places such as shopping centres and at work places. In a recently published study, extending the cervical cancer screening programme to the work place was suggested as a way to increase the number of eligible women having cervical cancer screening [18]. The same study concluded that women can be captured more easily at the work place and can also be traced for follow-ups. In Malaysia, women have been increasingly engaged in the workforce and their participation increased from 44.7% in 1995 to 47.3% in 2004 [36]; thus having Pap smear services available at the work place is a sensible suggestion. Our findings are supportive of the idea, as the participants also suggested introducing screening tests such as the Pap smear at their work place with minimal charges.

### Limitations

The study has several limitations. There was a focus on patients who have already been diagnosed with cancer and whose perceptions towards screening may differ from healthy individuals regarding knowledge of cancer and perceived effectiveness of cancer screening tests. The limitation of the funding restricted the study to only one hospital in Malaysia and therefore results of the research are not representative of the entire population. However, the results provide a glimpse of perceptions towards cancer screening from a population suffering from different types of cancer, disease progression, as well as from different cultural backgrounds. The study was conducted in one of the government hospitals which is usually approached by low to middle income groups, thus cancer screening practice might be better among patients from high income groups considering costs as a barrier to cancer screening.

## Conclusions

This qualitative exploratory study investigated the beliefs and experiences of cancer patients regarding cancer screening. The study identified important determinants of cancer patients’ beliefs towards cancer screening. Participants who had heard of cancer screening before the diagnosis never perceived themselves as being at risk of getting cancer. Lack of awareness about cancer screening tests and their benefits were identified as barriers to screening. The results have helped to identify barriers to knowledge acquisition, such as the language used in the dissemination of information.

## Competing interests

The authors declare that they have no competing interests.

## Authors’ contributions

MF conducted the field work and drafted the manuscript. MAH, AKS and AAS conceived and supervised the project. MAF, FS, NH, HA helped in analysis and manuscript review. All authors read and approved the final manuscript.

## Pre-publication history

The pre-publication history for this paper can be accessed here:

http://www.biomedcentral.com/1471-2458/13/48/prepub
